# Supplementary material for: Feasibility of integrating survivors of stroke into cardiac rehabilitation: A mixed methods pilot study
Source: PLoS One. 2021 Mar 29;16(3):e0247178. doi: 10.1371/journal.pone.0247178 (PMC8007047; doi:10.1371/journal.pone.0247178)
Supplement: S1 Checklist — (PDF) [file pone.0247178.s001.pdf]

CONSORT 2010 checklist of information to include when reporting a randomised trial*

Section/Topic	Item No	Checklist item	Reported on page No
**Title and abstract**			
	1a	Identification as a randomised trial in the title	N/A
	1b	Structured summary of trial design, methods, results, and conclusions (for specific guidance see CONSORT for abstracts)	2
**Introduction**			
Background and objectives	2a	Scientific background and explanation of rationale	3-5
	2b	Specific objectives or hypotheses	5
**Methods**			
Trial design	3a	Description of trial design (such as parallel, factorial) including allocation ratio	5
	3b	Important changes to methods after trial commencement (such as eligibility criteria), with reasons	none
Participants	4a	Eligibility criteria for participants	6
	4b	Settings and locations where the data were collected	5
Interventions	5	The interventions for each group with sufficient details to allow replication, including how and when they were actually administered	7-9
Outcomes	6a	Completely defined pre-specified primary and secondary outcome measures, including how and when they were assessed	9-10 fig 1 [illegible]
	6b	Any changes to trial outcomes after the trial commenced, with reasons	–
Sample size	7a	How sample size was determined	n/a - feasibility safety/protocol 5
	7b	When applicable, explanation of any interim analyses and stopping guidelines	
Randomisation:			
Sequence generation	8a	Method used to generate the random allocation sequence	n/A
	8b	Type of randomisation; details of any restriction (such as blocking and block size)	N/A
Allocation concealment mechanism	9	Mechanism used to implement the random allocation sequence (such as sequentially numbered containers), describing any steps taken to conceal the sequence until interventions were assigned	n/A
Implementation	10	Who generated the random allocation sequence, who enrolled participants, and who assigned participants to interventions	N/A
Blinding	11a	If done, who was blinded after assignment to interventions (for example, participants, care providers, those	N/A

		assessing outcomes) and how	
	11b	If relevant, description of the similarity of interventions	/
Statistical methods	12a	Statistical methods used to compare groups for primary and secondary outcomes	9-11
	12b	Methods for additional analyses, such as subgroup analyses and adjusted analyses	✓
**Results**			
Participant flow (a diagram is strongly recommended)	13a	For each group, the numbers of participants who were randomly assigned, received intended treatment, and were analysed for the primary outcome	fig 1
	13b	For each group, losses and exclusions after randomisation, together with reasons	fig 1
Recruitment	14a	Dates defining the periods of recruitment and follow-up	5
	14b	Why the trial ended or was stopped	
Baseline data	15	A table showing baseline demographic and clinical characteristics for each group	table 2
Numbers analysed	16	For each group, number of participants (denominator) included in each analysis and whether the analysis was by original assigned groups	/
Outcomes and estimation	17a	For each primary and secondary outcome, results for each group, and the estimated effect size and its precision (such as 95% confidence interval)	n/A this paper
	17b	For binary outcomes, presentation of both absolute and relative effect sizes is recommended	/
Ancillary analyses	18	Results of any other analyses performed, including subgroup analyses and adjusted analyses, distinguishing pre-specified from exploratory	~~32-3i~~
Harms	19	All important harms or unintended effects in each group (for specific guidance see CONSORT for harms)	32-34
**Discussion**			
Limitations	20	Trial limitations, addressing sources of potential bias, imprecision, and, if relevant, multiplicity of analyses	40-41
Generalisability	21	Generalisability (external validity, applicability) of the trial findings	41
Interpretation	22	Interpretation consistent with results, balancing benefits and harms, and considering other relevant evidence	36-40
**Other information**			
Registration	23	Registration number and name of trial registry	5
Protocol	24	Where the full trial protocol can be accessed, if available	OSF data 5
Funding	25	Sources of funding and other support (such as supply of drugs), role of funders	

*We strongly recommend reading this statement in conjunction with the CONSORT 2010 Explanation and Elaboration for important clarifications on all the items. If relevant, we also recommend reading CONSORT extensions for cluster randomised trials, non-inferiority and equivalence trials, non-pharmacological treatments, herbal interventions, and pragmatic trials. Additional extensions are forthcoming: for those and for up to date references relevant to this checklist, see www.consort-statement.org.

Consolidated criteria for reporting qualitative studies (COREQ): 32-item checklist

No	Item	Guide questions/description		
**Domain 1: Research team and reflexivity**				
Personal Characteristics				
1.	Interviewer/facilitator	Which author/s conducted the interview or focus group?	interview	pg 10
2.	Credentials	What were the researcher's credentials? *E.g. PhD, MD*	PhD, PT	title page
3.	Occupation	What was their occupation at the time of the study?	PhD student PT	pg 7
4.	Gender	Was the researcher male or female?	female	pg10
5.	Experience and training	What experience or training did the researcher have?		pg10
Relationship with participants				
6.	Relationship established	Was a relationship established prior to study commencement?	no	

No	Item	Guide questions/description	
7.	Participant knowledge of the interviewer	What did the participants know about the researcher? e.g. *personal goals, reasons for doing the research*	provided in consent
8.	Interviewer characteristics	What characteristics were reported about the interviewer/facilitator? e.g. *Bias, assumptions, reasons and interests in the research topic*	pg 11
**Domain 2: study design**			
Theoretical framework			
9.	Methodological orientation and Theory	What methodological orientation was stated to underpin the study? *e.g. grounded theory, discourse analysis, ethnography, phenomenology, content analysis*	pragmatic general pg10
Participant selection			
10.	Sampling	How were participants selected? *e.g. purposive, convenience, consecutive, snowball*	Criterion pg10
11.	Method of approach	How were participants approached? E.*g. face-to-face, telephone, mail, email*	~~by phone~~ / face to face pg10

No	Item	Guide questions/description	
12.	Sample size	How many participants were in the study?	pg14 12
13.	Non-participation	How many people refused to participate or dropped out? Reasons?	pg14
Setting			
14.	Setting of data collection	Where was the data collected? *e.g. home, clinic, workplace*	Clinic pg 10
15.	Presence of non-participants	Was anyone else present besides the participants and researchers?	no
16.	Description of sample	What are the important characteristics of the sample? *e.g. demographic data, date*	nested sample table 2
Data collection			
17.	Interview guide	Were questions, prompts, guides provided by the authors? Was it pilot tested?	yes on OSF website
18.	Repeat interviews	Were repeat interviews carried out? If yes, how many?	NO
19.	Audio/visual recording	Did the research use audio or visual recording to collect the data?	audio pg10

No	Item	Guide questions/description	
20.	Field notes	Were field notes made during and/or after the interview or focus group?	NO
21.	Duration	What was the duration of the interviews or focus group?	30min - 1hr.
22.	Data saturation	Was data saturation discussed?	n/a -
23.	Transcripts returned	Were transcripts returned to participants for comment and/or correction?	No
**Domain 3: analysis and findingsz**			
Data analysis			
24.	Number of data coders	How many data coders coded the data?	pg 10 2
25.	Description of the coding tree	Did authors provide a description of the coding tree?	pg 10 and OSF
26.	Derivation of themes	Were themes identified in advance or derived from the data?	pg 10 both
27.	Software	What software, if applicable, was used to manage the data?	NVivo pg 10
28.	Participant checking	Did participants provide feedback on the findings?	No

No	Item	Guide questions/description	
Reporting			
29.	Quotations presented	Were participant quotations presented to illustrate the themes / findings? Was each quotation identified? e.g. *participant number*	yes results multiple pages
30.	Data and findings consistent	Was there consistency between the data presented and the findings?	yes results
31.	Clarity of major themes	Were major themes clearly presented in the findings?	yes, themes structured
32.	Clarity of minor themes	Is there a description of diverse cases or discussion of minor themes?	yes minor themes provided
