# Supplementary material for: Feasibility of integrating survivors of stroke into cardiac rehabilitation: A mixed methods pilot study
Source: PLoS One. 2021 Mar 29;16(3):e0247178. doi: 10.1371/journal.pone.0247178 (PMC8007047; doi:10.1371/journal.pone.0247178)
Supplement: S1 File — (DOCX) [file pone.0247178.s002.docx]

# INTEGRATING SURVIVORS OF STROKE INTO CARDIAC REHABILITATION IMPROVES CARDIOVASCULAR ENDURANCE AND FUNCTIONAL STRENGTH

Elizabeth W. Regan, PhD, DPT^a^*, Reed Handlery, DPT^a^, Jill C. Stewart, PhD, PT^a^, Joseph L. Pearson, DrPH^b^, Sara Wilcox, PhD^a^, Stacy Fritz, PhD, PT^a^

*^a^ Exercise Science, University of South Carolina, 1300 Wheat Street Columbia, SC,29201 USA; ^b^ Health Promotion, Education and Behavior, University of South Carolina, 915 Greene Street Columbia, SC,29208 USA*

* Corresponding author: Elizabeth Regan [eregan@email.sc.edu](mailto:eregan@email.sc.edu) 704-609-2409

Key Words: Stroke; Cardiac Rehabilitation, Stroke Rehabilitation, Exercise

Short Title: Survivors of Stroke in Cardiac Rehabilitation

Word Count: 7319

## Abstract

Background:

Cardiac Rehabilitation (CR) is a structured exercise program prevalent in the U.S for people with cardiovascular disease that has been shown to increase cardiovascular endurance and improve quality of life. Despite similar cardiovascular risk factors, stroke is not among the covered diagnoses for CR.

The purpose of this study was to examine the participant impact of integrating survivors of stroke into an existing hospital-based CR program through measures of physical function and other health impacts, and through qualitative evaluation of participant perception.

Methods and Results: Subacute and chronic survivors of stroke were integrated into a standard 12-week, three sessions per week, CR program. Twenty-nine began, 24 completed, and 18 were available for six-month follow-up. Quantitative measures were compared pre- to post-program with t-test or equivalent, and pre to post to 6-month follow-up with ANOVA or equivalent. Semi-structured interviews were completed with 11 participants post-program. CR had significant impacts on cardiovascular endurance pre- to post-program with maintenance at six-month follow-up. The participants improved on the six-minute walk test by 61.92 meters (95% CI 33.99 – 89.84 meters) and maximum metabolic equivalents improved by a median of 3.6 (IQR 2.35). Five times sit to stand (functional strength) improved pre- to post-program by a median of 2.85 seconds (IQR 4.03 seconds). Qualitative findings highlight additional health improvements. Most participants (83% (15/18)) reported continued exercise at follow-up.

Conclusions: CR has the potential to improve cardiovascular endurance, health status and quality of life for survivors of stroke.

Clinical Trials Registration: URL: <http://www.clinicaltrials.gov>, NCTXXXXXXXX.

Clinical Perspective:

What is New?

- Including survivors of stroke into Cardiac Rehabilitation is an innovative way to reach an additional clinical population at risk for cardiovascular complications with structured exercise.
- The current study demonstrates the dosage and intensity of Cardiac Rehabilitation has the potential to improve cardiovascular endurance, health status and quality of life with some improvements persisting after six months.

What are the clinical implications?

- Medical and rehabilitation providers could have a new referral option to a standardized program shown to improve cardiovascular endurance, health status and quality of life for survivors of stroke.
- Currently, there are no standardized exercise programs for people post-stroke, so survivors are left to manage their activity on their own. Many face barriers and are generally inactive. Cardiac rehabilitation would provide an option for survivors over self-management.

## Introduction

Physical inactivity is a health concern for the majority of the seven million survivors of stroke in the United States (U.S.) who face increased risk for additional stroke and cardiovascular disease.^1^ Exercise can mitigate these risks, but survivors of stroke are not exercising; 58% fail to meet stroke guidelines for physical activity (PA).^2, 3^ While many survivors of stroke receive physical therapy immediately post-stroke, time barriers and an emphasis on functional activities limit cardiovascular exercise intervention.^4, 5^ As a result, survivors remain deconditioned after traditional rehabilitation, when they transition from one-on-one care with a physical therapist (PT) to self-directed individual activity.^6, 7^ The lack of appropriate community group exercise programs impedes continuation of supervised PA.^8^ Without guidance or knowledge on appropriate activity, most survivors of stroke do not continue to exercise or engage in PA post-rehabilitation.^9-11^ In addition to deconditioning and risk factor reduction, exercise may positively impact post-stroke depression, fatigue and community participation.^12-14^

Structured exercise programs offer an opportunity to break the cycle of inactivity, reduce future cardiac risk, perceived confidence and quality of life.^13-15^ In the U.S., Cardiac Rehabilitation (CR) is a structured and prevalent exercise program for people with cardiovascular diagnoses such as myocardial infarction.^16^ Participation in CR has been shown to increase functional exercise capacity and improve health related quality of life for traditional participants.^17-19^ Previous studies of cardiovascular training in survivors of stroke have demonstrated that they can safely perform aerobic exercise and achieve health benefits.^20-26^ Variation in dosage, staffing, and mode of activity impact the external validity of these studies;^20-26^ therefore, more knowledge is required to determine if benefits translate into existing CR programs. Research in Canada suggests the potential for integration of survivors of stroke into existing CR programs, however, the dosage and insurance climate differs from U.S. programs.^27-29^ Effectiveness in existing CR programs for survivors of stroke in the U.S. that follow Medicare guidelines has not been investigated. Evaluation of these programs is supported by the American Heart Association and the American Stroke Association.^14^

The primary aim of this study was to investigate the impact of an existing CR program for survivors of stroke through pilot measures for physical function (cardiovascular endurance, functional strength, walking speed), and for other health impacts (quality of life, balance confidence, depression, exercise habits). A secondary aim was to evaluate participant perception of program impact on physical function, health, and future exercise plans.

## Methods

The study was conducted at [health system in southeast] cardiac rehabilitation facility. A mixed methods design combined a single group, pre-post design, with a pragmatic qualitative inquiry of participant perception in order to enhance interpretation of results. All data and materials have been made publicly available at Open Science Framework.^30^

The project was approved by [health system] Institutional Review Board (IRB) and acknowledged by the [University] IRB. The study was a registered clinical trial through the United States National Library of Medicine (ClinicalTrials.gov ID: NCTXXXXXXX). Participation was voluntary and individuals were able to opt-out at any time. The program was free for study participants, with program costs ($237 per participant) covered by study grant funding. Participants provided informed consent and an authorization for use and disclosure of protected health information for research purposes.

Recruiting and Study Criteria:

Potential participants were consecutively recruited from [health system] rehabilitation clinicians, physicians, stroke team nurses and cardiac rehabilitation providers. Additionally, survivors of stroke were recruited directly from the community through stroke support groups and word of mouth referrals. Multiple referral targets were chosen to reflect likely general community program sources. Those who were interested were screened for eligibility by PI. (figure 1)

The following inclusion criteria were applied: (1) diagnosed with stroke at least 3 months prior (stroke diagnosis code and date provided on physician referral); (2) completed physical and occupational therapy, if applicable; (3) cleared by treating physician or nurse practitioner to participate; (4) demonstrated ability to walk at least 40 meters with or without an assistive device; (5) demonstrated ability to transfer from sit to stand without assistance; and (6) demonstrated ability to follow instructions and to communicate exertion, pain, and distress. Potential participants were excluded from the study for any of the following: (1) presence of a medical problem rendering exercise unsafe; (2) complaints of significant pain that interfered with movement; or (3) history of an additional, non-stroke, neurologic condition.

Once eligibility was determined, the PI (a PT) screened participants for safety and participants completed a demographic intake form and a battery of outcome measures. Screening (one-time mobility assessment at pre-program only) assessed range of motion, strength, balance limitations, and gait alterations. Initial determination of safety to participate was determined medically by the referring physician and from an independent mobility perspective by the study PI.

Program Procedures and Progression:

The mobility screening measures, and the pre-program outcome measures were shared with the CR staff to establish initial exercise intensity goals and modifications to the standard CR program. Participants were integrated into the standard CR program. Aside from modifications provided by the participant evaluation, the intervention did not differ from the standardized program. The program began with analysis to determine baseline levels of exercise intensity in METs based on participant’s six-minute walk test (6MWT) results. Target HR was estimated from resting HR and 6MWT completion HR. Target exercise rating of perceived exertion (RPE) levels were set from 11-14 (somewhat hard to hard) on a scale of 6-20.^31^ Activity plans were also individualized. Training sessions were three times a week for 12 weeks with a target of 31-50 minutes of moderate aerobic activity each session. Additional optional activities included strengthening, stretching, and/or relaxation. While components varied by session and individual, the general format was warm up, cardiovascular endurance activities (treadmill, recumbent step machine, recumbent bike, over ground walking), cooldown, and optional activities.

Progression in the program was determined by participant reported RPE. If RPE was consistently rated < 11, effort was increased to reach a rate of 14. Discontinuation of a session or the program was determined by standard health system protocols.

Weekly formal educational sessions were not adapted for survivors of stroke. Sessions were available to survivors of stroke participants but were not utilized.

At the end of the 12-week CR program, all participants were reassessed using the study outcome measures and an additional inquiry of participant’s post-program exercise plans. Completion of the program included all participants with final outcome measures available at post-program assessment.^32^

Six-months after the end of the CR program (follow-up), program completers were invited to return for the last outcome measure assessment which included an additional self-report of current exercise habits.

Outcome Measures:

Study PI administered outcome measures pre-program, post-program and six-months post-program (Table 1 and supplemental Table I). Maximum metabolic equivalents (METs) are a standard measure of exercise tolerance and functional capacity in CR programs.^33^ All remaining outcome measures have been validated in survivors of stroke.^34-40^

T**able 1.** Outcome Measures

| Outcome Measure | Assessment |
| --- | --- |
| Six-Minute Walk Test | - Cardiovascular Endurance; walking capacity; initial fitness in CR programs^18, 34^ - Measured as distance in meters |
| Five-Times Sit to Stand Test | - Functional Lower Body Strength^38, 41^ - Measured as seconds to complete |
| Ten-meter Walk Test | - Self-Selected and Fast Walking Speed^34, 42^ - Measured as meters/second |
| Maximum Metabolic Equivalents (METs) * | - Standard measure of exercise tolerance and functional capacity in CR programs.^33^ - Measured from 1 (very low) to 13 (very high) fitness. |
| Activities-Specific Balance Confidence Scale | - Self-Perception of Balance Confidence^37^ - Total score 0-100% Confidence - A score less than 67% indicates an increased risk of falls.^43^ |
| Stroke Impact Scale | - Impact of stroke on eight domains: mobility, participation, activities of daily living, hand function, strength, communication, emotion and memory/thinking.^35^ - Domain sub scores from 0% (significant impact) to 100% (no impact). |
| Short Self-Efficacy and Outcome Expectations for Exercise (SSEE and SOEE) | - Confidence to complete exercise behaviors such as exercising alone or through fatigue.^39^ - Exercise outcome expectations such as belief that exercise improves mood or improves endurance. ^39^ - Scores are 1 (low) to 5 (high) |
| Patient Health Questionnaire-9 | - Depression^40^ - Total score of 0-27 with categories of 0 (no depression), 1 to 9 (minimum to mild), 10 to 14 (moderate), and 15 to 27 (moderately severe to severe). |
| * Maximum Metabolic Equivalents were calculated at the initial visit and final visits as part of the standard program and were not re-assessed at six-month follow-up. | |

Outcome Measures Statistical Analysis:

Power analysis was conducted based on findings from a previous study with a similar population and exercise intervention.^44^ Calculations suggested 22 participants would provide 80% power to detect pre-post changes moderate in magnitude (effect size d = 0.56) in the 6MWT.

Participant demographic information and outcome measures were aggregated with means, medians and standard deviations calculated. Aggregate means and standard deviations for total number of sessions, session time and minimum and maximum RPE were calculated for fidelity of the program to contextualize outcome measure results. The outcome measure data for the full sample of completers pre-post program (n=24) were analyzed using a paired t-test or Wilcoxon Signed Rank Test (for those not normally distributed or ordinal variables). The alpha level was set at 0.01 due to multiple comparisons and the desire to minimize both type I and type II errors.^45^ Effect sizes (Cohen’s d) were generated. Finally, for the subset of the sample where six-month follow-up data were available (n=18), a repeated measures ANOVA or a Friedman’s test was completed for those measures found to be statistically different in the pre-post program comparison. Bonferroni adjustments were made to the ANOVA and Friedman’s Tests. Analysis was completed with IBM SPSS Statistics for Windows, version 26 (IBM Corp., Armonk, N.Y., USA).

Qualitative Methods and Analysis:

A pragmatic qualitative approach evaluated participant perspectives on program outcomes and future exercise plans.^46, 47^ Interview questions were developed based on study aims and framed by the World Health Organization’s International Classification of Function and Social Cognitive Theory.^48, 49^ The interview guide is provided in supplemental Table II.

The sampling plan was nested within the original sample and potential qualitative participants were identified sequentially as they completed the program. All participants who began the program and met qualitative eligibility requirements were invited to voluntarily participate. Participants who had previously participated in CR or had verbal communication limitations were ineligible for the qualitative portion. Participants in the qualitative portion of the study provided separate informed consent and received a $20 gift card as an incentive. Semi-structured interviews were conducted in a private room at the time of post-program outcome measures collection. Interviews were audio recorded, and transcribed verbatim. Questions were piloted in the first two interviews and revised slightly. The number of participant interviews were determined by maximal voluntary participation of those eligible in order to achieve saturation of themes.^50^ Field notes and addition of quantitative data added rigor.^51^

The researchers completed inductive thematic analysis using NVivo software (version 12, QSR International),^52^ with de-identified transcripts and observation notes. One researcher coded all interviews to phrases or sentences directly from the transcripts and structured observations. Results were reviewed with a second researcher. Both researchers then independently performed inductive categorizing of the open coding. A final thematic codebook was agreed upon. Each researcher updated independent coding to reflect the codebook. Data conflicting with primary themes were identified to present alternative viewpoints.^52^ Results were compared, and any discrepancies resolved together. Final coding was reviewed with a third researcher where naming conventions and minor alterations were made.

## Results

Of the 29 participants starting the program, there were 24 completers. Of the five non-completers, two dropped out, one was unable to be independent due to agitation/cognitive issues, one had an additional mild stroke and no longer met the inclusion criteria, and one had recurrent bronchitis. Eighteen of the 24 completers returned for six-month follow-up assessments (Figure 1). Six completers were lost to follow up, one declined and five were impacted by COVID-19 facility closure. Eleven of the thirteen eligible completers participated in the qualitative interviews.

Program participant demographics are presented in Table 2. The most common co-morbid health conditions included high blood pressure (83.3%), diabetes (41.7%), cardiovascular disease (41.7%), and arthritis (29.2%). The average number of sessions per completer was 25.25 (95% CI 22.91 – 27.92) with a range of 12-36 sessions. Participants averaged 38.93 (95% CI 36.54 – 41.31) exercise minutes per session and met RPE targets of 11 (light) to 14 (somewhat hard) with minimum RPE median of 11 (IQR 0.625) and maximum RPE median of 13 (IQR 1.00) across all sessions. There were no safety events related to exercise intensity and all participants met the prescribed RPE ranges.

Results of pre to post-program comparisons are presented in Table 3 and Table 4, and for pre-program, post-program and six-month post-program comparisons in Figure 2. Outcomes and qualitative themes are presented for (1) cardiovascular endurance, (2) other physical outcomes and general health, (3) emotional health, (4) exercise self-efficacy and outcomes expectations, and (5) post-program exercise.

**Table 2.** Demographics of Program Participants

| Gender, % (number) | Age, mean (SD) | Race / Ethnicity, % (number) | Type of Stroke, % (number) ^*^ | Time Since Stroke, mean (SD) | Initial 6MWT Distance Category, % (number) † | Initial SSWS, mean (SD) | Pre-Program Exercise Level,  % (number) |
| --- | --- | --- | --- | --- | --- | --- | --- |
| Completers (n=24) | | | | | | | |
| 79% (19) Male  21% (5) Female | 62.2 (12.4) years | 71% (17) White  25% (6) African American  4% (1) Asian | 65% (15) Ischemic  12.5% (3) Hemorrhagic  25% (6) Unknown | 29.7 (29.9) Months | 83.3% (20)  > 288m  16.7% (4)  < 288m | 1.17 (0.21) | 12.5% (3)  None  12.5% (3)  <1 x week  37.5% (9)  1-3 x week  37.5% (9)  > 3 x week |
| Non-Completers(n=5) | | | | | | | |
| 60% (3)  Male  40% (2)  Female | 68.4 (15.0) years | 60% (3) White  40% (2) African American | 40% (2) Ischemic  40% (2) Hemorrhagic  20% (1) Unknown | 37.0 (41.8) Months | 40% (2)  > 288m  40% (2)  < 288m  20% (1)  no 6MWT | 0.67 (0.28) | 40% (2)  None  0% (0)  <1 x week  60% (3)  1-3 x week  0% (0)  > 3 x week |

Abbreviation: SD, standard deviation; 6MWT, six-minute walk test; SSWS, self-selected walking speed; m/s, meters per second; m, meters;

^*^ Stroke type was self-report by participant

†^*^ six-minute walk test > 288m indicates community ambulator status.^42^

**Table 3.** Results Pre-Program to Post-Program: Paired T-Test Outcome Measures

| **Test** | **N** | **Mean (SD)**  **Pre** | **Mean (SD) Post** | **Mean (SD) Change** | **95% CI of Mean Change** | **t** | **df** | **Significance (p)** | **Effect Size (d)** |
| --- | --- | --- | --- | --- | --- | --- | --- | --- | --- |
| 6MWT (m) ^*^ | 24 | 397.80 (119.23) | 459.71 (118.46) | ↑ 61.92 (66.13)† | 33.99 – 89.84 | 4.587 | 23 | <0.001‡ | 0.94 |
| FWS (m/s) § | 23 | 1.50 (0.42) | 1.59 (0.50) | ↑ 0.09 (0.18) | 0.02 –  0.17 | 3.167 | 22 | 0.019 |  |

Abbreviation: 6MWT, Six Minute Walk Test; m, meters; FWS, Fast Walking Speed; m/s, meters per second.

^*^ Higher distance indicates an improvement in score.

† Greater than the minimal detectable change for stroke of 31m.^34^

‡Statistically significant changes.

§ Higher number indicates a faster walking speed

**Table 4.** Results Pre-Program to Post-Program: Wilcoxon Signed Rank Test Outcome Measures

| **Test** | **N** | **Median**  **(IQR) Pre** | **Median (IQR) Post** | **Median (IQR) Change** * | **Z** | **Sig. (p)** |  |
| --- | --- | --- | --- | --- | --- | --- | --- |
| **Cardiovascular Endurance Measures** | | | | | | |  |
| SIS-Mobility  (0-100%) | 24 | 72.22 (31.25) | 77.78 (29.17) | ↑ 6.94 (11.11) † | 2.665 | 0.008 ‡ |  |
| MET Max  (1-low to 13 high) | 24 | 2.95 (0.88) | 6.00 (3.00) | ↑3.6 (2.35) | 4.199 | <0.001 ‡ |  |
| **Physical Function measures** | | | | | | |  |
| FTSS (s)  (lower score-better | | 23 | 14.42 (11.14) | 12.2 (6.47) | ↓ 2.85 (4.03) \|\| | -3.528 | < 0.001‡ |
| SSWS (m/s) (higher score-faster) | 23 | 1.16 (0.34) | 1.18 (0.38) | ↑ 0.02 (0.16) | 1.095 | 0.274 |  |
| ABC Score  (% Confidence) | 24 | 73.44 (35.28) | 86.38 (21.48) | ↑ 1.78 (14.61) | 1.686 | 0.092 |  |
| SIS-Physical  (0-100%) | 24 | 62.50 (37.50) | 75.00 (37.50) | - 0.00 (18.75) | 1.350 | 0.177 |  |
| **Quality of Life measures (Stroke Impact Scale-other subscales)** | | | | | | |  |
| SIS-Mood  (0-100%) | 24 | 77.78 (29.17) | 86.11 (20.14) | ↑ 4.17 (13.19) | 1.869 | 0.062 |  |
| SIS-Memory  (0-100%) | 24 | 78.57 (37.50) | 82.14 (27.68) | - 0.00 (16.96) | 2.076 | 0.038 |  |
| SIS-Communication  (0-100%) | 24 | 87.50 (50.00) | 83.93 (31.25) | - 0.00 (13.39) | 1.623 | 0.105 |  |
| SIS-ADLs  (0-100%) § | 24 | 90.00 (31.25) | 90.00 (16.90) | ↑ 2.50 (9.38) | 2.425 | 0.013 |  |
| SIS-Hand  (0-100%) | 24 | 85.00 (43.75) | 92.50 (40.00) | - 0.00 (10.00) | 1.002 | 0.316 |  |
| SIS-Participation  (0-100%) | 24 | 70.31 (53.91) | 76.56 (39.06) | ↑ 3.13 (21.09) | 1.976 | 0.048 |  |
| SIS-Recovery  (0-100%) | 24 | 80.00 (15.00) | 82.50 (15.00) | ↑5.00 (10.00) | 1.715 | 0.086 |  |
| **Self-Efficacy Measures (exercise and outcomes expectations)** | | | | | | |  |
| SSEE  (1-low to 5-high) | 22 | 4.20 (1.19) | 4.50 (0.69) | ↑ 0.25 (1.06) | 2.023 | 0.043 |  |
| SSOE  (1- low to 5- high) | 22 | 4.00 (0.60) | 4.20 (1.60) | ↑ 0.20 (0.65) | 2.397 | 0.017 |  |

Abbreviation: IQR, interquartile range; Sig, significance; SIS, Stroke Impact Scale; MET Max, Metabolic Equivalents Maximum; FTSS, Five-Times Sit to Stand; s, seconds; SSWS, Self-Selected Walking Speed; m/s, meters per second; ABC-Activities Specific Balance Confidence; SIS, Stroke Impact Scale; ADLs, Activities of Daily Living; SSEE, Short Self-Efficacy for Exercise; SOEE, Short Outcomes Expectations for Exercise.

* ↑ indicates improvement, ↓ indicates decline, and - indicates no change.

† Change is greater than the clinically important difference rate of 4.5%.^53^

|| Change is greater than the 1.14s minimal detectable change for survivors of stroke.^54^

‡ Statistically significant changes <0.01

§ SIS-ADLs change score distribution was not symmetrical, so a Sign test was completed instead of Wilcoxon-Signed Rank Test.

Cardiovascular Endurance:

The 6MWT, the Stroke Impact Scale (SIS) mobility subscale and maximum METs measured cardiovascular endurance. The 6MWT, the primary outcome measure for aerobic and walking capacity, improved by 61.92 m (95% CI 33.99 – 89.84 m) pre-post program with a large effect size (0.94), which is greater than the minimal detectable change of 34 m for survivors of stroke (Table 3). ^34, 55^ Improvements in 6MWT distances were maintained at six-month follow-up results (Figure 2a).

The SIS-Mobility subscale had a statistically significant median improvement post-program of 6.94%, which is greater than the clinically important difference of 4.5% (Table 4).^53^ However, comparisons including the six-month follow-up did not find a statistically different change over time (p=0.057).

Maximum METs progressed with a median difference of 3.6 (IQR 2.35) from the beginning of the program (first session) to the end of the program (final session at week 12) (Table 4).

Qualitative themes related to endurance included improved stamina, improved stair climbing, and needing less rest breaks during activity. For some, improved endurance impacted their physical activity tolerance, and they were able to do more of what they enjoy.

Participant 11: “I think that, because of improving my stamina and my endurance, that has um, helped me in other things. So, um it, it’s allowed me to do a little bit more dancing, and a little bit and, and not have to constantly be resting as much… ”

Participant 15: “Um, yes it's...think...just walking and uh, uh just general um physical activities and I think...I don't wanna over say it, it but ah I have to think that, ah, it's improved my every day, ah, activity tolerance.”

Other Physical and General Health Outcomes:

Other physical outcome measures included strength, walking speed, stability and balance, and general health impacts. The Five Times Sit to Stand (FTSS) test measured lower extremity strength which improved by a median of 2.85 (IQR 4.03) seconds (Table 4). FTSS gains remained at six-month follow-up (Figure 2b).^54^ Participants maintained but did not improve their walking speed, ABC Scale or SIS-Physical Subscale scores pre- to post-program. The proportion of participants in the highest fall risk category (ABC Scale <67%) was 33.3% (n=8) pre-program, and 20.8% (n=5) post-program. A few participants noticed balance improvements, with qualitative themes noting improved reaction times, and better balance confidence. Several participants noted improvements in their walking often related to improved stability, balance and strength.

Participant 18: “My reflexes are getting quicker. I can, I can look both ways quicker on the crosswalk, and I can run across the street and I can read the car coming at me quicker.”

General health outcome measures included the remaining Stroke Impact Scale subscales (SIS-ADLs, SIS-Hand, SIS-Communication, SIS-Memory, SIS-Participation and SIS-Recovery (overall self-rated stroke recovery)); all without statistically significant changes. A few participants noted health changes not covered above; themes included weight loss/improved physical appearance, positive medication changes, and improved awareness of importance of health.

Participant 3 “… I have been thinking about my health and how to live the best life that I can and I think this program has encouraged this thinking on my part.”

Interviewer: “Okay, and what are you thinking you need to do to live the best life? Like are you thinking about changes you need to make?”

Participant 3: “Well I got a referral for speech therapy and I am doing that now, and I am not sure that would have occurred to me before. And um I think the eating has been better.”

Emotional Health:

Several study outcomes measured emotional health: the PHQ-9, the SIS-Mood subscale and an analysis of qualitative interviews. The SIS-Mood subscale did not have statistically significant changes pre-post program. Twenty-three participants had initial PHQ-9 depression screen scores at pre-program: 11.5% (n=2) in the moderately severe-severe depression categories, 17.4% (n=4) in moderate depression category, 69.6% (n=16) in the minimum-mild depression category and 4.3% (n=1) in the no-depression category. These depression category proportions remained mostly unchanged at post-program where 24 participant scores were available with 11.5% (n=2) in the moderately severe-severe depression categories, 11.5% (n=2) in moderate depression category, 70.8% (n=17) in the minimum-mild depression category and 12.5% (n=3) in the no-depression category. While a few participants noted no changes to mood or outlook as a result of the program, many participants noted improvements in emotional health including reduced depression, contributions to a positive attitude, and improved self-perception. Participants noted a new or renewed sense of enthusiasm for exercise or for engaging in activities and feeling more confident about their abilities.

Participant 12: “Overall experience was, it was, it was kind of life changing. Kind of life saving. Um, definitely haven't been nearly as depressed as I was before I came in here. Not at all. Um, and that doesn't just have to do with [life change]. It was, it was night and day difference. After about two weeks of being in here, it was night and day difference. From being really dark and, and in a really bad way. Um, really depressed, and, and trying to almost, uh, not really sure what to do with it, and I kind of starting, getting faith again, hope, feeling good, wanting to take care of myself, and, and just being happy.”

Exercise Self Efficacy, Exercise Outcome Expectations and Post-Program Exercise:

Participants had high initial scores for both the Short Self-Efficacy for Exercise (SSEE) (median 4.20 out of 5) and the Short outcome expectation of Exercise (SOEE) (median 4 out of 5) indicating their confidence to exercise was high and that they anticipated benefits from exercise. Changes post-program were not statistically significant (p>0.01).

Post-Program Exercise Plans:

All completers had plans to continue exercise post-program. Plans included continuing at CR through the self-pay maintenance program, participating in group-based exercise classes, joining a gym for aerobic and strength activities, doing exercise at home, and working with a personal trainer.

At six-month follow-up, 83.3% (15/18) of participants reported engaging in exercise at least once a week, 44.4% (8/18) with a frequency of one to three times a week, and 38.9% (7/18) with a frequency of greater than three times a week. Reported activities included walking (50%), gym-strengthening (22.2%), gym-aerobic (50%), home-aerobic (33.3%), home-strengthening (11.1%), group exercise (22.2%), and other (22.2%) which included swimming, yardwork, horseback riding, and running.

## Discussion

After participation in CR, survivors of stroke made improvements in cardiovascular endurance, functional strength and perceived mobility. Improvements in cardiovascular endurance and functional strength were maintained at the six-month follow-up suggesting the possibility of lasting changes. Qualitative results confirmed endurance and mobility improvements and highlighted additional improvements in emotional health. Improvements occurred regardless of self-reported prior activity levels. Previous exercise experience combined with high levels of exercise self-efficacy may have been a driver for initial participation.

Cardiovascular Endurance:

*Survivors improve cardiovascular endurance*

Survivors of stroke integrated into CR demonstrated improvements in cardiovascular endurance. The 6MWT test improvements suggest better community walking status and real world walking capability.^42, 56^ The importance of this increase in capacity is especially important to survivors of stroke who have mobility impairments which result in a higher energy cost for walking.^56^ The 6MWT improvements were maintained at six-month follow-up, supporting maintenance of gains after CR. The 61.92 m change pre-post program was greater than a pooled mean change of 53.3 m from a recent meta-analysis of aerobic programs for stroke survivors with similar dosage to cardiac rehabilitation.^57^ Maximum METs had a median increase of 3.6 METs pre-post program. These changes are important measures of overall health. A meta-analysis by Kodama et al. found that in healthy individuals, for each one MET increase in exercise capacity, all-cause mortality was reduced by 13% and incidence of coronary heart disease and cardiovascular disease was reduced by 15%.^58^ Similar results have been found for traditional CR participants.^59^ The SIS-mobility scale measures participant perception of home and community mobility capabilities, and improvement pre-program to post-program corroborate the link between capacity and participation. These results were not maintained at six-month follow-up, however. The addition of social support from other participants and from staff during the CR program may have impacted the SIS-Mobility results which did not continue in the follow-up period.^60^ Qualitative themes of improved stamina impacting participant’s daily activities support the quantitative findings of improved cardiovascular endurance and overall mobility.

Other Physical and General Health Outcomes:

*Survivors improve functional strength*

Lower extremity strength improved pre- to post-program and was maintained at six-month follow-up. In addition to measuring strength, the FTSS test has speed and control components, and functional correlates.^54, 61, 62^ For survivors of stroke, taking longer to complete the FTSS test correlates with lower bilateral knee flexor strength and increased risk of falls.^54, 62^ For geriatric populations, which often include survivors of stroke, a slower time is predictive of less independence in activities of daily living within three years.^63^ Collectively, improvements in cardiovascular endurance and strength support the positive health and fitness benefits of integration of survivors of stroke into U.S. based CR programs.

*Emotional Health and Self-Efficacy for Exercise had Ceiling Effects and Mixed Results*

The initial scores measuring this construct indicated low initial depression in the sample (73.9% with no-mild depression on the PHQ-9) and higher initial mood (median of 77.78% on the SIS-Mood subscale), leaving little room for change. Qualitative results suggest that participation in CR may impact emotional health for individuals, and this is supported by existing research.^64-67^ The qualitative themes related to emotional health were participants finding renewed self-confidence and sense of self. Higher self-esteem is known to positively impact self-perception of identity after stroke.^65-67^ A qualitative study by Erikson et al. found that finding a positive new self-identity after stroke was tied to engaging with others through meaningful activities which a program like CR can provide.^66^

Self-efficacy for exercise and outcome expectation for exercise scores were high at pre-program suggesting good to excellent confidence in exercise abilities (SSEE, median 4.2) and belief in benefits of exercise (SOEE, median 4.0). With a maximum score of 5 on both the SSEE and the SOEE, achieving significant changes was difficult due to a ceiling effect. The high initial scores in this sample may be related to the importance of having self-efficacy and positive outcome expectations to commit to structured exercise programs.^68, 69^ All of the completers had concrete plans for continued exercise at the completion of the program. At six-months, the majority were still active, suggesting that 12 weeks may be long enough to build habits for maintained activity. However, improved exercise habit results require more investigation, as this sample had a high proportion of participants with high self-efficacy for exercise and some exercise experience prior to the program, both key drivers of on-going physical activity in survivors of stroke.^69^

Study Limitations:

Study limitations include the use of a single group pilot design at a single CR program, and lack of diversity among participants. While a diverse sample of mobility impairments, gender, age and racial/ethnic diversity was desired, most participants were Caucasian men with few mobility limitations and relatively high initial fitness levels. Future studies for survivors with less functional mobility may require further screening and expansion of CR protocols. Additionally, future studies can expand to multiple health system sites and utilize a randomized control trial design with recruiting plans targeting participants with specific characteristics.

## Conclusion

CR for survivors of stroke had a positive impact on cardiovascular endurance and functional strength. CR also influenced participant’s perception of their home and community mobility, their walking capability, and their emotional health. Improvements in METs correspond to reduced risk for mortality and cardiovascular disease. Despite similar cardiovascular risk factors to traditional CR participants and potential health benefits from participation, stroke is not among the covered diagnoses for CR services in the United States. Findings support the use of CR programs for survivors of stroke after rehabilitation to improve endurance, health status and quality of life. Further investigations can confirm findings and explore integrating survivors of stroke as a standard of care.

**Funding**

University of South Carolina (USC) Behavioral-Biomedical Interface Program (NIGMS/NIH-T32 2T326M081740-11A1)**,** 2019 American Heart Association (AHA) Pre-Doctoral Fellowship, 2018 American Physical Therapy Association Health Policy and Administration Research Grant, 2019 USC Support to Promote Advancement of Research and Creativity Grant, AHA Grant 15SDG24970011, Promotion of Doctoral Studies (PODS)–Level I Scholarship from the Foundation for Physical Therapy Research; the Arnold Fellowship from the Arnold School of Public Health, USC.

**Disclosures:** None

**References:**

1. Benjamin EJ, Muntner P, Bittencourt MS. Heart disease and stroke statistics-2019 update: A report from the american heart association. *Circulation*. 2019;139:e56-e528

2. Hardie K, Hankey GJ, Jamrozik K, Broadhurst RJ, Anderson C. Ten-year risk of first recurrent stroke and disability after first-ever stroke in the perth community stroke study. *Stroke*. 2004;35:731-735

3. Butler EN, Evenson KR. Prevalence of physical activity and sedentary behavior among stroke survivors in the united states. *Topics in stroke rehabilitation*. 2014;21:246-255

4. Jette DU, Latham NK, Smout RJ, Gassaway J, Slavin MD, Horn SD. Physical therapy interventions for patients with stroke in inpatient rehabilitation facilities. *Physical Therapy*. 2005;85:238-248

5. Buntin MB, Colla CH, Deb P, Sood N, Escarce JJ. Medicare spending and outcomes after post-acute care for stroke and hip fracture. *Medical care*. 2010;48:776

6. Blennerhassett JM, Levy CE, Mackintosh A, Yong A, McGinley JL. One-quarter of people leave inpatient stroke rehabilitation with physical capacity for community ambulation. *Journal of stroke and cerebrovascular diseases : the official journal of National Stroke Association*. 2018

7. MacKay-Lyons MJ, Makrides L. Longitudinal changes in exercise capacity after stroke. *Archives of Physical Medicine and Rehabilitation*. 2004;85:1608-1612

8. Boyne P, Billinger S, MacKay-Lyons M, Barney B, Khoury J, Dunning K. Aerobic exercise prescription in stroke rehabilitation: A web-based survey of united states physical therapists. *Journal of neurologic physical therapy: JNPT*. 2017;41:119

9. Robinson CA, Shumway-Cook A, Ciol MA, Kartin D. Participation in community walking following stroke: Subjective versus objective measures and the impact of personal factors. *Physical therapy*. 2011;91:1865-1876

10. Michael KM, Allen JK, Macko RF. Reduced ambulatory activity after stroke: The role of balance, gait, and cardiovascular fitness. *Archives of physical medicine and rehabilitation*. 2005;86:1552-1556

11. Kono Y, Kawajiri H, Kamisaka K, Kamiya K, Akao K, Asai C, et al. Predictive impact of daily physical activity on new vascular events in patients with mild ischemic stroke. *International Journal of Stroke*. 2015;10:219-223

12. Hebert D, Lindsay MP, McIntyre A, Kirton A, Rumney PG, Bagg S, et al. Canadian stroke best practice recommendations: Stroke rehabilitation practice guidelines, update 2015. *International journal of stroke : official journal of the International Stroke Society*. 2016;11:459-484

13. Woodman P, Riazi A, Pereira C, Jones F. Social participation post stroke: A meta-ethnographic review of the experiences and views of community-dwelling stroke survivors. *Disability and rehabilitation*. 2014;36:2031-2043

14. Billinger SA, Arena R, Bernhardt J, Eng JJ, Franklin BA, Johnson CM, et al. Physical activity and exercise recommendations for stroke survivors a statement for healthcare professionals from the american heart association/american stroke association. *Stroke*. 2014;45:2532-2553

15. Patterson S, Ross-Edwards B. Long-term stroke survivors' needs and perceptions of an exercise maintenance model of care. *International Journal of Therapy and Rehabilitation*. 2009;16:659-669

16. Curnier DY, Savage PD, Ades PA. Geographic distribution of cardiac rehabilitation programs in the united states. *Journal of Cardiopulmonary Rehabilitation and Prevention*. 2005;25:80-84

17. Humen D, Higgins G, Unsworth K, Prior P, Massel D, Suskin N. A cost analysis of event reduction provided by a comprehensive cardiac rehabilitation program. *Canadian Journal of Cardiology*. 2013;29:S156

18. Bellet RN, Adams L, Morris NR. The 6-minute walk test in outpatient cardiac rehabilitation: Validity, reliability and responsiveness—a systematic review. *Physiotherapy*. 2012;98:277-286

19. Anderson L, Taylor RS. Cardiac rehabilitation for people with heart disease: An overview of cochrane systematic reviews. *International journal of cardiology*. 2014;177:348-361

20. Marzolini S, Tang A, McIlroy W, Oh PI, Brooks D. Outcomes in people after stroke attending an adapted cardiac rehabilitation exercise program: Does time from stroke make a difference? *Journal of Stroke and Cerebrovascular Diseases*. 2014;23:1648-1656

21. Boss H, Van Schaik S, Deijle I, de Melker E, van den Berg B, Scherder E, et al. Safety and feasibility of post-stroke care and exercise after minor ischemic stroke or transient ischemic attack: Motives & moveit. *NeuroRehabilitation*. 2014;34:401-407

22. Lennon O, Carey A, Gaffney N, Stephenson J, Blake C. A pilot randomized controlled trial to evaluate the benefit of the cardiac rehabilitation paradigm for the non-acute ischaemic stroke population. *Clinical Rehabilitation*. 2008;22:125-133

23. Biasin L, Sage MD, Brunton K, Fraser J, Howe JA, Bayley M, et al. Integrating aerobic training within subacute stroke rehabilitation: A feasibility study. *Physical Therapy*. 2014;94:1796-1806

24. Cuccurullo SJ, Fleming TK, Kostis WJ, Greiss C, Gizzi MS, Eckert A, et al. Impact of a stroke recovery program integrating modified cardiac rehabilitation on all-cause mortality, cardiovascular performance and functional performance. *American journal of physical medicine & rehabilitation*. 2019;98:953-963

25. Lennon O, Blake C. Cardiac rehabilitation adapted to transient ischaemic attack and stroke (crafts): A randomised controlled trial. *Bmc Neurology*. 2009;9

26. Kirk H, Kersten P, Crawford P, Keens A, Ashburn A, Conway J. The cardiac model of rehabilitation for reducing cardiovascular risk factors post transient ischaemic attack and stroke: A randomized controlled trial. *Clinical rehabilitation*. 2014;28:339-349

27. Marzolini S. Integrating individuals with stroke into cardiac rehabilitation following traditional stroke rehabilitation: Promoting a continuum of care. *Canadian Journal of Cardiology*. 2018

28. Tang A, Marzolini S, Oh P, McIlroy WE, Brooks D. Feasibility and effects of adapted cardiac rehabilitation after stroke: A prospective trial. *Bmc Neurology*. 2010;10

29. and UCfM, Medicaid Services. Your medicare coverage: Cardiac rehabiltiation programs.2020

30. Regan E. Integrating stroke survivors into cardiac rehabilitation. 2020

31. Dunbar CC, Robertson RJ, Baun R, Blandin MF, Metz K, Burdett R, et al. The validity of regulating exercise intensity by ratings of perceived exertion. *Medicine & Science in Sports & Exercise*. 1992

32. Santiago de Araújo Pio C, Varnfield M, Sarrafzadegan N, Beckie TM, Babu AS, Baidya S, et al. Promoting patient utilization of outpatient cardiac rehabilitation: A joint international council and canadian association of cardiovascular prevention and rehabilitation position statement. 2019

33. Shiran A, Kornfeld S, Zur S, Laor A, Karelitz Y, Militianu A, et al. Determinants of improvement in exercise capacity in patients undergoing cardiac rehabilitation. *Cardiology*. 1997;88:207-213

34. Flansbjer U-B, Holmbäck AM, Downham D, Patten C, Lexell J. Reliability of gait performance tests in men and women with hemiparesis after stroke. *Journal of rehabilitation medicine*. 2005;37:75-82

35. Lai S-M, Studenski S, Duncan PW, Perera S. Persisting consequences of stroke measured by the stroke impact scale. *Stroke*. 2002;33:1840-1844

36. Nadarajah M, Mazlan M, Abdul-Latif L, Goh H. Test-retest reliability, internal consistency and concurrent validity of fatigue severity scale in measuring post-stroke fatigue. *European journal of physical and rehabilitation medicine*. 2016

37. Botner EM, Miller WC, Eng JJ. Measurement properties of the activities-specific balance confidence scale among individuals with stroke. *Disability and rehabilitation*. 2005;27:156-163

38. Mong Y, Teo TW, Ng SS. 5-repetition sit-to-stand test in subjects with chronic stroke: Reliability and validity. *Archives of physical medicine and rehabilitation*. 2010;91:407-413

39. Shaughnessy M, Resnick BM, Macko RF. Reliability and validity testing of the short self-efficacy and outcome expectation for exercise scales in stroke survivors. *Journal of Stroke and Cerebrovascular Diseases*. 2004;13:214-219

40. Williams LS, Brizendine EJ, Plue L, Bakas T, Tu W, Hendrie H, et al. Performance of the phq-9 as a screening tool for depression after stroke. *Stroke*. 2005;36:635-638

41. Beninato M, Portney LG, Sullivan PE. Using the international classification of functioning, disability and health as a framework to examine the association between falls and clinical assessment tools in people with stroke. *Physical Therapy*. 2009;89:816-825

42. Fulk GD, He Y, Boyne P, Dunning K. Predicting home and community walking activity poststroke. *Stroke*. 2017;48:406-411

43. Lajoie Y, Gallagher S. Predicting falls within the elderly community: Comparison of postural sway, reaction time, the berg balance scale and the activities-specific balance confidence (abc) scale for comparing fallers and non-fallers. *Archives of gerontology and geriatrics*. 2004;38:11-26

44. Langhammer B, Stanghelle JK, Lindmark B. An evaluation of two different exercise regimes during the first year following stroke: A randomised controlled trial. *Physiotherapy Theory and Practice*. 2009;25:55-68

45. Feise RJ. Do multiple outcome measures require p-value adjustment? *BMC medical research methodology*. 2002;2:8-8

46. Patton MQ. *Qualitative research and evaluation methods*. Sage; 2015.

47. Morgan DL. Pragmatism as a paradigm for social research. *Qualitative Inquiry*. 2014;20:1045-1053

48. Bandura A. Social cognitive theory of self-regulation. *Organizational behavior and human decision processes*. 1991;50:248-287

49. World Health Organization. International classification of functioning, disability and health (icf). 2017

50. Morse JM. Determining sample size. 2000

51. Tong A, Sainsbury P, Craig J. Consolidated criteria for reporting qualitative research (coreq): A 32-item checklist for interviews and focus groups. *International Journal for Quality in Health Care*. 2007;19:349-357

52. Braun V, Clarke V. Using thematic analysis in psychology. *Qualitative research in psychology*. 2006;3:77-101

53. Lin K, Fu T, Wu C, Wang Y, Liu J, Hsieh C, et al. Minimal detectable change and clinically important difference of the stroke impact scale in stroke patients. *Neurorehabilitation and neural repair*. 2010;24:486-492

54. Kwong PW, Ng SS, Chung RC, Ng GY. Foot placement and arm position affect the five times sit-to-stand test time of individuals with chronic stroke. *BioMed research international*. 2014;2014

55. Eng JJ, Dawson AS, Chu KS. Submaximal exercise in persons with stroke: Test-retest reliability and concurrent validity with maximal oxygen consumption. *Archives of Physical Medicine and Rehabilitation*. 2004;85:113-118

56. Ribeiro JA, Oliveira SG, Thommazo-Luporini LD, Monteiro CI, Phillips SA, Catai AM, et al. Energy cost during the 6-minute walk test and its relationship to real-world walking after stroke: A correlational, cross-sectional pilot study. *Physical therapy*. 2019;99:1656-1666

57. Regan EW, Handlery R, Beets MW, Fritz SL. Are aerobic programs similar in design to cardiac rehabilitation beneficial for survivors of stroke? A systematic review and meta&#x2010;analysis. *Journal of the American Heart Association*. 2019;8:e012761

58. Kodama S, Saito K, Tanaka S, Maki M, Yachi Y, Asumi M, et al. Cardiorespiratory fitness as a quantitative predictor of all-cause mortality and cardiovascular events in healthy men and women: A meta-analysis. *Jama*. 2009;301:2024-2035

59. Martin B-J, Arena R, Haykowsky M, Hauer T, Austford LD, Knudtson M, et al. Cardiovascular fitness and mortality after contemporary cardiac rehabilitation. *Mayo Clinic Proceedings*. 2013;88:455-463

60. Cott CA, Wiles R, Devitt R. Continuity, transition and participation: Preparing clients for life in the community post-stroke. *Disability and rehabilitation*. 2007;29:1566-1574

61. Lord SR, Murray SM, Chapman K, Munro B, Tiedemann A. Sit-to-stand performance depends on sensation, speed, balance, and psychological status in addition to strength in older people. *The Journals of Gerontology Series A: Biological Sciences and Medical Sciences*. 2002;57:M539-M543

62. Buatois S, Perret-Guillaume C, Gueguen R, Miget P, Vançon G, Perrin P, et al. A simple clinical scale to stratify risk of recurrent falls in community-dwelling adults aged 65 years and older. *Physical Therapy*. 2010;90:550-560

63. Zhang F, Ferrucci L, Culham E, Metter EJ, Guralnik J, Deshpande N. Performance on five times sit-to-stand task as a predictor of subsequent falls and disability in older persons. *Journal of aging and health*. 2013;25:478-492

64. Eng JJ, Reime B. Exercise for depressive symptoms in stroke patients: A systematic review and meta-analysis. *Clin Rehabil*. 2014;28:731-739

65. Lapadatu I, Morris R. The relationship between stroke survivors’ perceived identity and mood, self-esteem and quality of life. *Neuropsychological rehabilitation*. 2019;29:199-213

66. Erikson A, Karlsson G, Tham K. Living with the long-term consequences 11–13 years after stroke: A phenomenological study. *Journal of rehabilitation medicine*. 2016;48:847-852

67. Morris JH, Oliver T, Kroll T, Joice S, Williams B. Physical activity participation in community dwelling stroke survivors: Synergy and dissonance between motivation and capability. A qualitative study. *Physiotherapy*. 2017;103:311-321

68. Dixon G, Thornton EW, Young CA. Perceptions of self-efficacy and rehabilitation among neurologically disabled adults. *Clinical rehabilitation*. 2007;21:230-240

69. Morris JH, MacGillivray S, McFarlane S. Interventions to promote long-term participation in physical activity after stroke: A systematic review of the literature. *Archives of Physical Medicine and Rehabilitation*. 2014;95:956-967

**Figure Captions**

**Figure 1:** Study Flowchart.

**Figure 2:** Changes Over Time (**A**) Improvements over time in mean six-minute walk test distance (6MWT), n=18 (p=0.001); **p=0.002, *p=0.013. 6MWT means (SD) Pre-program 403.18m (108.34), post-program 474.85m (101.49), six-month follow up 469.70m (26.17). Error bars are standard deviation. (**B**) Improvements over time in the five-times sit to stand (FTSS) test time in pairwise comparisons, n=17 (p<0.001); **p<0.001. Faster time indicates a better score. Boxplots show median, interquartile range, minimum and maximum. FTSS median ( IQR) pre-program 14.23s (7.15), post-program 11.88s (5.44), six-moth follow up 10.75s (6.92). One participant used one upper extremity for support to rise to standing during testing.
