# Supplementary material for: Feasibility of integrating survivors of stroke into cardiac rehabilitation: A mixed methods pilot study
Source: PLoS One. 2021 Mar 29;16(3):e0247178. doi: 10.1371/journal.pone.0247178 (PMC8007047; doi:10.1371/journal.pone.0247178)
Supplement: Attachment — Submitted filename: PLos one review .docx [file pone.0247178.s003.docx]

Thank you for inviting me to review this exciting manuscript entitled feasibility of integrating survivors of stroke into cardiac rehabilitation: a mixed-methods pilot study. This study examined the feasibility of integrating survivors of stroke into an existing CR. I commend the authors for taking this massive project and the ability to synthesis this project with multiple outcomes into a single mixed-method manuscript. Generally, the manuscript would improve if the authors address some of my concern below. In part, the manuscript was well written. The authors should expressly state their research questions and the corresponding study design that answered the question. For instance, from table 1, it seems that the qualitative part of the mixed method answered only recruitment, uptake and retention; acceptability and safety, why quantitative answers recruitment, uptake, retention; adherence and fidelity; safety and effectiveness. Since the effectiveness of the CR program has been reported elsewhere, can the authors consider only focusing on exploring only the program characteristics as it regards to recruitment, uptake, retention, adherence and fidelity, and safety? I had conducted this review systematically using various headings, and I have provided examples (when necessary).

**Title:**

Consider adding- "Hospital-based" in the topic. e.g. Feasibility of integrating survivors of stroke into hospital-based, cardiac rehabilitation: a mixed-methods pilot study.

**Abstract**

- Can the authors consider separating methods from the result?
- I would suggest that the authors revise the conclusion as it did not align with the result presented. Throughout the result section, there was no evidence about participants meeting the standard dosage and intensity goals.

**Introduction**

- Line 27, can the authors explain a bit more the behaviour they were referring to in this statement. As it is written, the reader might not know the behaviour they were referring to.
- Lines 36, can the authors be specific with the references. For instance, only 24% of time at > 40% maximum heart rate (MHR) in one (10), and 4.8% of the time at > 60% MHR in another (11).
- The authors did well by mentioning studies primarily outside the US that implemented cardiac rehab programs for survivors of stroke. A summary of these studies and the differences with the authors' hospital-based CR would be useful.
- Can the authors provide some distinction of community-based cardiac rehab programs and hospital-based (inpatient or outpatient) cardiac rehab programs?; this would provide a more robust rationale and justify why understanding the dosage and other parameters to ensure continuing of CR after discharge is essential.

**Methods**

- Thank you for providing a reference for the health system CR. A brief description of the health system CR program, specifically on the Phases-as non-cardiac diagnoses were allowed to participate in the program after Phase II, would be helpful for the reader.
- Rationales for inclusion criteria would be helpful or reference. For instance, why a diagnosis of stroke at least three months prior, ability to walk at least 40 meters with or without an assistive device etc.
- Can the authors provide specifically the component of the standard CR and the specific modifications? As it is written in lines 92-109, the reader is not sure if it is a standard program or the modified program.
- What does the standardised intake form consist of? This will align adequately when the authors described the demographics. For instance, the standardised intake form consists of the age, sex and number of PA activity before enrolling into the program, type of stroke, affected area of the stroke, etc.
- I would suggest the authors check the equator network to consider which reporting guideline is appropriate for their study. For instance, there should be a heading for a program description, which the authors described the health system CR program, its standard components and its modifications. I believe all of these are in part located in across the manuscript, but reporting guideline would help organise the information for an easy read.
- The authors should develop a qualitative methodology. What kind of qualitative paradigm, descriptive or interpretative. What is the criteria to participate in the qualitative criteria, was it criterion-based purposive sampling or convenience sampling.
- In Table 1, did the authors consider asking a question about patients perception about the benefits of the program?
- What guideline was used in the structured observation? What exactly was the PI observing? It would be great to have this information for the reader to understand how it added to the qualitative rigour. Does this mean that data for the qualitative component of this feasibility pragmatic study were: single individual face-to-face or telephone interview and PI's observation? If yes, it should be stated clearly. Does the PI observation purely for triangulation or to provide for context to the individual interviews? These should be clear in the method section of the qualitative study.
- Was there any need for any form of member checking for the qualitative component? How do the authors' reflexivity influences data analysis?
- Generally, as I understand that the authors used both inductive and deductive thematic approaches to analyse their data, but they failed to state how they combined these two distinct approach. At what level was inductive occur and deductive? Does it mean the PI first round of open coding is considered inductive, and the use of codebook is considered deductive? Deductive, the two coders map the themes emerged under the broad categories. If yes, these should be very explicit in the data analysis method.w I would suggest authors look for frameworks for program uptake, barriers and facilitators to guide their deductive analysis.
- Did the authors consider if the type of stroke (ischemic or hemorrhagic or others could have any impact on the 1) recruitment, uptake and retention, (2) adherence and fidelity, (3) acceptability, (4) safety, and (5) effectiveness? as they did not consider that in their method section. What is the severity of their stroke? Does this matter or not? This information will provide context to the study to ensure applicability in a similar context (external validity).

**Result:**

- Table 2: Can the authors also provide a range for the time since stroke, as it will give us additional information? How was the pre-program exercise level calculated? This may be in the body of the text, but Tables should be a stand-alone, and a foot description of how it was calculated will have the reader?
- Reporting individual deficits (lines 202-205) might not be the best approach. Can the authors consider reporting individuals with single deficits (weakness= 13, walking= 10..... ) and those with two or more deficits (n=). These will give us more information in terms of deficits. We know that multiple deficits, either physical, cognitive or environmental, can influence adherence. Did the authors consider this?
- I believe that the authors quoted verbatim the participants responses in the manuscript. Providing grammatical corrections will improve the readability of the participant's quote. For instance, participant 4 (under competing time demands), can be written as: So [am] not being able to [be] here [attend the CR program] three times a week um, work came into play. Alternatively, better still choose a more clear quote to support any theme emerged.
- Lines 245 -248 is confusing. The first sentence seems like the authors' perception. While the first sentence is significant, it would be great if the authors present the result and then provide their perspective as to why people enrolled in the program in the discussion section of the manuscript.
- The theme "disinterest in gym exercise", is it more of disinterest in structured gym exercise...
- Line 312, is it all participants that were informally exposed to nutrition and exercise consultation?
- Based on table 1, there is no qualitative interview topic for adherence and fidelity. Can the authors align which area the qualitative or quantitative data answered? If both data answered all areas, the authors should also state it and then ensure that all outcomes for quantitative data and interview questions reflect in Table 1.
- Thank you for providing the type of equipment the participants did not like. While this is informative for clinicians, did the participants stated why they prefer some equipment and why they did not prefer some. Authors should provide reasons why participants prefer one thing vs the other(see line 393), and throughout the manuscript.
- Thank you for providing the statement on line 366. This type of statement should be stated in the method section of the manuscript.
- The three safety severe events occurred outside CR was not associated with the CR. if it is related to the CR, they should provide context on how it was related to the events. This is a situation a qualitative interview will provide context on how the three serious safety events are related to the CR program. I would advise authors only to report safety events that are related to the CR program. We cannot control what happens outside the CR program or can infer the impact of the CR during the CR program period.
- How is (a) the number and type of mobility impairment, and (b) mobility and safety consultations,- safety issues? Does it mean the participants developed these mobility impairments when they started the program or what? Can the authors provide context on this. How were the safety events assessed, self-reported or via observation as the author mentioned?

**Discussion**

- Line 557 stated on CR was the right intensity was not supported by the study found. Rather participants stated that they liked the frequency, and no question of intensity was asked. Can the authors consider rewording this statement?
